# Frequency of eNOS polymorphisms in the Colombian general population

**DOI:** 10.1186/1471-2156-11-54

**Published:** 2010-06-20

**Authors:** Norma C Serrano, Luis A Díaz, Juan P Casas, Aroon D Hingorani, Daniel Moreno de Lucca, María C Páez

**Affiliations:** 1 Biomedical Research Centre, Health Sciences Faculty, Universidad Autónoma de Bucaramanga, Bucaramanga, Postal 1642, Colombia; 2 Department of Epidemiology and Public Health, University College London, London, WC1E 6BT, UK; 3 Department of Epidemiology and Population Health, London School of Hygiene and Tropical Medicine (LSHTM), London, WC1E 7HT, UK; 4 Centre for Clinical Pharmacology, Department of Medicine, University College London, London, WC1E 6BT, UK

## Abstract

**Background:**

Nitric oxide (NO) synthesized by endothelial cells is known to be a potent vasodilator. It has been suggested that polymorphisms in endothelial nitric oxide synthase (eNOS) can affect the response of the vascular endothelium to increased oxidative stress. The objective of the present study was to determine the presence of G894T (rs1799983), intron-4 (27-bp TR) and -T786C (rs2070744) polymorphisms in the eNOS gene among the Colombian general population.

**Results:**

Genotype and allele frequencies showed significant differences in their distribution. White, black and mixed populations were in HW equilibrium for the variants in 27-bp TR- and rs1799983, but the black population was in HW disequilibrium for rs2070744 (p < 0.001). Allele "T" of rs1799983 polymorphisms was more common in the white population (26,5%) than the others, while allele "C" of rs2070744 polymorphisms had a similar frequency in all populations, and the allele 4a from 27-bp TR was more frequent in the black population (26,2%) than the others. Similar differences were found when genotypes were analyzed.

**Conclusion:**

The findings suggest that there is a substantial difference in the distribution of eNOS polymorphisms between different ethnic groups. These results could aid the understanding of inter-ethnic differences in NO bioavailability, cardiovascular risk, and response to drugs.

## Background

Cardiovascular disease (CVD) comprises a group of complex diseases that are highly prevalent worldwide, but show marked variation according to ethno geographical distribution [1]. Environmental and genetic factors contribute to ethnic variation and susceptibility to CVD; interethnic differences in disease patterns can be due to environmental and/or genetic factors [2].

Nitric oxide (NO) plays a pivotal role in the regulation of cardiovascular homeostasis. This highly reactive molecule is produced in endothelial cells and platelets by endothelial NO synthase (eNOS); it maintains basal vasodilator tone, inhibits platelet aggregation, attenuates leukocyte adhesion to the endothelium, and modulates smooth muscle proliferation [3]. Given the importance of NO for cardiovascular function, polymorphisms that alter eNOS activity or its regulation are likely candidates for studies examining the genetic variability of vascular disease [4]. Three clinically relevant polymorphisms in endothelial eNOS have been associated with many CVDs: single nucleotide polymorphisms (SNPs), one in the promoter region (T-786C) and the other in exon 7 (G894T), and the variable number of tandem repeats (VNTR) in intron 4 [5].

Inconsistent associations between these eNOS variants and CVD have been found. One possible explanation for the contrasting results is that such association studies normally focus on the contributions of a single polymorphism to a specific clinical condition and may not detect modest effects [6]. Furthermore, population stratification, as a consequence of ethnic diversity, could decrease the reproducibility of association studies and dilute the power of case-control studies designed to identify genetic risk factors for a disease, particularly when ethnic variations in allele frequencies in cases and controls are not matched [7]. Reliable information regarding the distribution of eNOS variants in different ethnic populations is required in order to improve the design of association studies.

The Colombian population is very heterogeneous as result of extensive inter-ethnic crosses between people from different continents such as Europeans, Africans and autochthonous Amerindians [6]. This study was conducted to examine the distribution of three clinically relevant polymorphisms found in the eNOS gene in five Colombian ethnic groups, and to estimate the haplotype frequency in these groups.

## Results

Nine hundred and seventeen people were interviewed and 890 individuals were included in this study; 27 were excluded (15 that were selected for convenience and 12 who did not agree to give a blood sample). Table 1 shows the frequencies of genotypes and alleles according to the region of origin, demonstrating a considerable difference in the distribution of eNOS polymorphisms among different cities. The distributions of genotypes for each polymorphism were in HW equilibrium, with the exception of the Tamá Paez Amerindian population in which the rs1799983 and rs2070744 genotypes were in HW disequilibrium (p < 0.001 in both cases).

**Table 1:** Genotype and allelic frequencies by place of recruitment

		Place of recruitment						p-value
			
Frequencies		*Bucaramanga (n = 140)*	*Pasto (n = 136)*	*Cartagena (n = 135)*	*Quibdó (n = 169)*	*Pereira (n = 141)*	*Neiva (n = 169)*	
***Genotype frequencies***								
rs1799983	G, G	0.443	0.676	0.704	0.820	0.667	0.540	<0.001
	G, T	0.457	0.309	0.267	0.180	0.312	0.410	
	T, T	0.100	0.015	0.030	-	0.021	0.050	

rs2070744	T, T	0.571	0.684	0.563	0.777	0.525	0.511	<0.001
	T, C	0.379	0.287	0.363	0.187	0.418	0.439	
	C, C	0.050	0.029	0.074	0.036	0.057	0.050	

27-bp TR	4b, 4b	0.914	0.890	0.615	0.453	0.688	0.741	
	4b, 4a	0.086	0.096	0.311	0.403	0.298	0.209	
	4a, 4a	-	0.070	0.030	0.043	0.014	0.014	
	4a, 4c	-	-	0.015	0.022	-	-	<0.001
	4b, 4c	-	0.070	0.030	0.065	-	0.022	
	4b, 4y	-	-	-	0.014	-	0.014	
	4c, 4c	-	-	-	-	-	-	

***Allelic frequencies***								
rs1799983	G	0.675	0.838	0.837	0.910	0.823	0.752	<0.001
	T	0.325	0.162	0.163	0.090	0.177	0.248	

rs2070744	T	0.761	0.827	0.752	0.871	0.734	0.741	<0.001
	C	0.239	0.173	0.248	0.130	0.266	0.259	

27-bp TR	4b	0.957	0.938	0.781	0.694	0.837	0.871	<0.001
	4a	0.043	0.059	0.200	0.255	0.163	0.112	
	4c	-	0.004	0.019	0.043	-	0.011	
	4y	-	-	-	0.007	-	0.007	

Table 2 presents genotype and allele frequencies according to ethnic background and demonstrates significant differences in their distribution. Owing to differences in the frequencies of alleles and genotypes between both Amerindian populations studied (Tamá Paez and Emberá), each group was analysed separately. White, black and mixed populations were in HW equilibrium for the variants in 27-bp TR and rs1799983, but the black population was in HW disequilibrium for rs2070744 (p < 0.001).

**Table 2:** Genotype and allelic frequencies by ethnic group

		Ethnic group					
Frequencies		*White (n = 323)*	*Black (n = 145)*	*Mixed (n = 357)*	*Emberá (n = 30)*	*Tamá Paez (n = 30)*	p-value
***Genotype frequencies***							
rs1799983	G, G	0.536	0.828	0.658	0.933	0.567	<0.001
	G, T	0.396	0.172	0.319	0.067	0.433	
	T, T	0.068	-	0.022	-	-	

rs2070744	T, T	0.570	0.759	0.574	0.967	0.533	<0.001
	T, C	0.372	0.200	0.381	0.033	0.467	
	C, C	0.059	0.041	0.045	-	-	

27-bp TR	4b, 4b	0.820	0.434	0.737	0.967	0.700	<0.001
	4b, 4a	0.164	0.421	0.221	0.033	0.200	
	4a, 4a	0.009	0.041	0.017	-	-	
	4a, 4c	-	0.021	0.060	-	-	
	4b, 4c	0.003	0.069	0.017	-	-	
	4b, 4y	0.003	0.014	0.003	-	0.033	
	4c, 4c	-	-	-	-	0.067	

***Allele frequencies***							
rs1799983	G	0.735	0.914	0.824	0.967	0.783	<0.001
	T	0.265	0.086	0.176	0.033	0.217	

rs2070744	T	0.754	0.859	0.773	0.983	0.767	<0.001
	C	0.246	0.142	0.227	0.017	0.233	

27-bp TR	4b	0.904	0.686	0.859	0.983	0.817	
	4a	0.093	0.262	0.130	0.017	0.100	<0.001
	4c	0.002	0.045	0.010	-	0.067	
	4y	0.002	0.007	0.001	-	0.017	

There were considerable differences in allelic variants between the ethnic groups analysed. Variant 298T (rs1799983) was more common in the white population (26.5%) than the others. The C variant (rs2070744) had a similar frequency in the white, mixed and Tamá Paez Amerindians, was less frequent in the black population (14.2%), and had the lowest frequency in Emberá Amerindians (1.7%).

**Figure 1:**
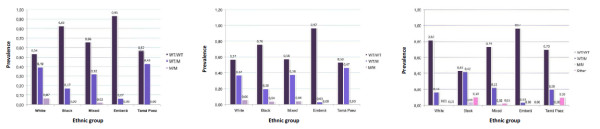
**Prevalence of genotypes among ethnic groups**. **Panel A: rs1799983 (G894T)**. W: wildtype (allele G), M: mutation (allele T) **B: rs2070744 (T**^-^**786C)**. W: wildtype (allele T), M: mutation (allele C) **C: 27-bp TR (VNTR) **W: wildtype (allele 4b), M: mutation (allele 4a), Other: genotype minor frequency (4a/4c, 4b/4c, 4b/4c, 4b/4y, 4c/4c).

As figure 1 demonstrates, the allele rare "a" of the 27-bp TR variable was most frequent in the black population (26.2%) and had the lowest frequency in the Emberá Amerindians (1.7%). The frequencies of the rarer alleles (c, y) were less than 5% among all the populations (Table 2), except for allele "c" among the Tama Paez Amerindians, which had a frequency of 6.7%.

Similar differences were found when genotypes were analysed; the homozygous rs1799983 variant was more common among the white population than the mixed population (6.8% and 2.2%, p = 0.004), and it was absent in the black population (p = 0.001) and Amerindians (p = 0.06). The CC of rs2070744 variant was more frequent in the white population, while black and mixed populations had a similar frequency (table 2), and it was absent among Amerindians. However, these differences were not statistically significant.

Genotype 4aa of 27-bp TR was more frequent among the black and the mixed populations. However, the only significant difference was between the black and white populations (p = 0.048, figure 1c). Is important to note the high heterozygosity for this marker in blacks (0.42), which doubles the frequency value reported in mixed and exceeds with statistically significance the value in the other populations.

The frequencies of the haplotypes considered were calculated for the different ethnic groups (table 3). The most common haplotype in all ethnic groups was *G,T,4b*, which combines the wild-type variants of all three polymorphisms. This wild-type haplotype was present at the highest frequency in the Emberá Amerindians (93.3%).

**Table 3:** Estimated haplotype frequency by ethnic group

Haplotype			Ethnic group				
**rs1799983**	**rs2070744**	**27-bp TR**	***White (n = 323)***	***Black (n = 145)***	***Mixed (n = 357)***	***Emberá (n = 30)***	***Tamá Paez (n = 30)***

G	T	4b	0.6017	0.5366	0.6108	0.9333	0.6638
G	T	4a	0.0362	0.2070	0.0749	0.0167	
G	C	4b	0.0457	0.0763	0.0803	0.0167	
G	C	4a	0.0487	0.0431	0.0487		0.0529
G	T	4c		0.0439	0.0053		0.0500
G	T	4y	0.0016	0.0069			0.0167
G	C	4c	0.0016		0.0803		
T	C	4b	0.1422	0.0220	0.0874		0.1167
T	C	4a	0.0080		0.0054		0.0047
T	C	4c			0.0001		0.0167
T	C	4y			0.0014		
T	T	4b	0.1145	0.0513	0.0801	0.0333	0.0362
T	T	4a		0.0120	0.0012		
T	T	4c		0.0009	0.0009		

The second most common haplotype was *G,T,4a*, which included the variable 4a from 27-bp TR and the wild-type allele for the other polymorphisms. This haplotype was present at a higher frequency among the black population (20.7%) than the other groups analysed (Table 3). Two haplotypes with high frequencies in the white population were *T,C,4b *(14.2%) and *T,T,4b *(11.4%), both of which include the minor allele frequency "T" to the rs1799983 variant; the difference in frequency compared with the other populations was statistically significant (p < 0.001). The haplotypes *Glu,C,4y *and *T,T,4y *were not detected.

**Figure 2:**
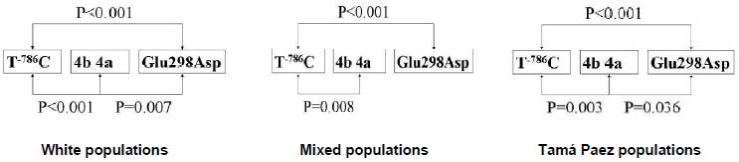
**Linkage disequilibrium by ethnic group**. Likelihood disequilibrium by pair-wise combinations of polymorphisms in three ethnic groups.

Linkage analysis between each pair of combinations demonstrated specific associations between ethnic groups. There is linkage disequilibrium (LD) between the white and mixed populations for the three polymorphisms but not between the black and Emberá populations. LD was demonstrated among the Tamá Paez Amerindians, between rs2070744 and rs1799983 and between rs2070744 and 27-bp TR-. The relationship among the three ethnic groups in which LD exists is presented in figure 2.

## Discussion

There were marked differences in the distribution of the eNOS variants, the frequency of the haplotypes examined and in the association between the variables in the Colombian ethnic groups analyzed.

Two studies have been published showing inter-ethnic differences in the frequencies of these three eNOS polymorphisms, which are considered clinically relevant to CVD. The first study concerned Caucasian, African American and Asian populations residing in the USA [6]; the second was carried out in white and black populations from Brazil [8]. A third study, analysing 4055 Japanese individuals, analyzed two of these polymorphisms [9]. The frequency of the variable T298 (rs1799983) among the white Colombian population and reported in other study was similar. However, the analysis of the Black population shows significant differences; we found no homozygous individuals for this allele in the Colombian black population, while in USA African Americans this frequency was 10.0%, and homozygous individuals were present in Brazilian black and Japanese populations. The inter-ethnic and geographical differences could explain the high incidence and severity of hypertension among African Americans [10,11].

Homozygous CC from the rs2070744 variant (promoter region) has similar frequencies in black populations in various studies, less than 5%. However, we found significant differences among white populations; it is less frequent in the Colombian (5.9%) than the USA (16.0%) and Brazilian (15.0%) populations.

In the 27-bp TR variant, the genotype "aa" was present at lower frequency in the black populations analysed than the other study done in a Latin American populations (4.1% vs. 12.5%).

The variants 4c and 4y were not present in Caucasian North American and Brazilian populations, but were present at a low frequency (0.2%) in the white Colombian population. An allele that defined like "4d" that presents three 27pb repetitions, one more than allele "4a", has been reported in an Italian population but not in Latin American populations and we found no evidence for it in the present study [12].

Comparing the results of this study with previous reports suggests that there are inter-ethnic differences for various polymorphisms of eNOS, based on the geographical origin of the ethnic group analysed [13].

The most common haplotype for each of the ethnic groups analysed in this study includes the wild-type variants (Glu,T,4b) that have a high frequencies among Emberá Amerindians (93.3%). A recent study concerning a Latin American population presented similar results, the highest frequency being present in Amerindian tribes from the Brazilian Amazon compared with black and white populations [14].

The next two most frequent haplotypes in the white Colombian population are the T allele from the rs1799983 variant and the C allele from the rs2070744 variant, both of which are in linkage disequilibrium in this ethnic group. These findings suggest that the high cardiovascular risk attributed to the rs2070744 SNP (promoter region) [15] and the rs1799983 variant [16] in Caucasians could be due to the combined effect of these variants on the expression of eNOS. Together with the low frequency of variable 4a in this population, these results suggest that the C variable from the promoter region could be more relevant than the 4a variant in Caucasians. In support of this hypothesis, Metzger et al. demonstrated a lower prevalence of this haplotype in healthy white males with a lower production of nitric oxide [17].

The rs1799983 SNP is the only analyzed variant found in a coding region of the gene; several studies have shown the functional effect of this substitution. The first approximation was achieved through *in vitro *studies undertaken by Teasuro et al. These authors used human cardiac and endothelial cells transfected with the polymorphic variant T298 and found that the resulting protein was more susceptible to cleavage by internal proteases and hence rapidly inactivated, reducing the production of NO [18]. Individuals homozygous for T-rs1799983 could suffer a rapid loss of the mature protein due to cleavage, which would translate into a lower production and bioavailability of NO [19].

However, subsequent study demonstrate that the exchange of glutamate against aspartate at position 298 in human eNOS does not alter the catalytic function of protein. Thus, this study suggested that the 298 G to T polymorphism affects endothelial NO synthesis through the intracellular targeting and/or changes in the phosphorylation state of eNOS in vivo [20]. In support of this, it was demonstrated that healthy pregnant women with T-rs1799983 had a 21% reduction in flux-mediated vasodilatation, a response dependent on NO, compared with women without the polymorphism [21]. A study analyzed these three variants in Mexican Americans in relation to the risk of cardiovascular-renal disease and demonstrated that variations in T-rs1799983 are directly linked to cardiovascular disease, altering the generation of NO [22].

Other studies have shown that the black Colombian population has a higher heterozygosity to the 27-bp TR variant than white or Amerindians populations. This variant has been associated with an increased risk of developing cardiovascular [21] and renal [22] diseases in African Americans. Results regarding the biological role of this variant have been contradictory; some reports, such as that by Zhang et al., indicate that carriers of the mutated variants have low plasma levels of NO. This could be caused by the presence of the 27-nt microRNA acting as an endogenous molecule for the feedback regulation of eNOS expression in human endothelial cells [23,24]. However, these results have not been reproduced in all studies [25,26]. This variant could act with another functional variant in the regulatory region of eNOS as a marker of linkage disequilibrium. In the Colombian population studied herein, this variant was found in linkage disequilibrium with G894T and T-786C in the white and mixed populations, and with T-786C in the Tamá Paez native population. In the last one, Amerindian populations, present endogamy with relative frequency, that can be cause of this LD, and in this case was difficult exclude consanguineous subjects.

The results of this study demonstrate the frequency of the alleles, genotypes and related haplotypes from three eNOS polymorphisms in the Colombian population. These polymorphisms are relevant as markers for cardiovascular risk in different populations worldwide in addition to patients with preeclampsia [27,17]. The ethnic differences suggest that cases and controls should be matched by ethnicity in future genetic association studies. In addition, the homozygosity frequency for rare alleles in the three polymorphisms were different among ethnic groups, and could serve as a basis for defining sample sizes when investigating significant differences between populations and estimating the effects of such polymorphisms.

## Conclusions

The present findings and those previously reported suggest that there is a substantial difference in the distribution of eNOS genetic variants between different ethnic groups. These results could help understanding of inter-ethnic differences in NO bioavailability, CVD risk and responses to medications.

## Methods

### Sample

The study was descriptive, transversal and population-based, carried out in urban cities from six Colombian regions identified as having different ethnical configurations (Bucaramanga, Cartagena, Pasto, Quibdó, Pereira and Neiva). In addition, it included members of two Amerindian communities (Emberá from Chocó, near Quibdó; and Tamá Paez from Huila, near Neiva). The study was evaluated and approved by the ethical review board from the Biomedical Research Centre at the Universidad Autónoma de Bucaramanga. All participants signed an informed consent form.

Individuals from urban settings were selected at random in a three-stage process to include at least 140 individuals from each city. All stages involved single sampling using independent random number lists; in the first step, a sample of blocks from urban maps were selected; in the second step, houses were selected in a similar way; the third stage selected one individual from each selected household. The participants recruited were healthy males and females aged between 18 and 44 years, who were born and lived in the studied region.

A survey was conducted that concerned demographic data, ethnic and familial backgrounds. Each person was classified as white, black, Amerindian or mixed according to skin colour, characteristics of hair and facial features. Each person gave his/her approval for the ethnic classification that was awarded, or gave their own ethnic classification. All participants were invited to give a peripheral blood sample for DNA extraction.

### DNA extraction and genotyping

Blood was drawn from the antecubital vein into a blood tube containing EDTA and samples were stored at -20°C until DNA extraction. DNA was extracted using the QIAamp DNA blood minikit (QIAGEN^©^, Hilden, Germany). The G894T (rs1799983) polymorphism was genotyped by polymerase chain reaction-restriction fragment length polymorphism (PCR-RFLP) using the following primer pairs: 5'AGG AAA CGG TCG CTT CGA CGT GCT G 3' and 5'CCC CTC CAT CCC ACC CAG TCA ATC C 3' [18]. PCR was performed for 35 cycles in a volume of 30 μL. Denaturation was carried out at 95°C, annealing at 63°C, and a final extension at 72°C, for 45 s. Ten microliters of each PCR product (151 bp) was subjected to restriction digestion with 2 U *Dpn *II, which cleaves the PCR product into fragments of 49 and 101 bp in the presence of the T allele (corresponding to T298). The digested samples were resolved by electrophoresis.

Genotypes for intron 4, 27-bp TR polymorphism were determined by PCR using the primers 5'-AGG CCC TAT GGT AGT GCC TTT-3' and 5'-TCT CTT AGT GCT GTG GTC AC-3'. PCR was performed for 35 cycles in a volume of 40 μL. The PCR reaction mixture was heated to 94°C for four min for denaturation and underwent 35 cycles at 94°C for 30 s for denaturation, at 63°C for 30 s for annealing, and at 72°C for one min for extension. Final extension was conducted at 72°C for five min. PCR products were resolved by electrophoresis. Fragments of 447, 420, 393 y 339 bp correspond to the eNOS alleles 4c, 4b, 4a and 4y, which defined the presence of six, five, four and two 27-bp repeats [6,12].

The T-786C (rs2070744) variant in the 5'-flanking region was assessed by PCR amplification using the primers 5'-TGG AGA GTG CTG GTG TAC CCC A-3' and 5'-GCC TCC ACC CCC ACC CTG TC-3' with the same temperature cycles described for polymorphism in intron 4 [17]. The amplified products were digested with *Msp I *for overnight at 37°C, producing fragments of 140 and 40 bp for the wild-type allele, or 90, 50, and 40 bp in case of polymorphism variants. These fragments were analyzed by electrophoresis. All genotyping was conducted anonymously. For quality control, 10% of the samples were subjected to repeat PCR and genotyping, and no discrepancies were detected.

### Statistical analysis

Information was typed in duplicate in Epi info 2000 version 3.2. Using this software, the genotype and allele frequencies for each polymorphism in the ethnic groups and regions were calculated. The statistical significance of the differences was calculated using a chi square test, and a value of p < 0.05 was used as the significance threshold.

The Hardy-Weinberg Equilibrium (HWE) of each of the three polymorphisms for each region and ethnic group was calculated with Arlequin software. In addition, we estimated the frequencies of the possible haplotypes and the presence of linkage disequilibrium in each ethnic group. This software uses maximum likelihood processes in all estimates.

## Authors' contributions

NCS conceived the study, carried out the molecular genetic studies, participated in its design and coordination and helped to draft the manuscript. LAD conceived and designed the study and performed the statistical analysis. JPC participated in the analysis and interpretation of data and helped to draft the manuscript. ADH participated in the analysis and interpretation of data and helped to draft the manuscript. MCP carried out the molecular genetic studies and participated in the analysis and interpretation of data. DML helped to draft the manuscript.

All authors read and approved the final manuscript.
